# 

**Published:** 1892-04-02

**Authors:** 


[Registered at the General Post Office as a Newspaper for transmission in the United Kingdom and Abroad.]

WITH EXTRA NURSING SUPPLEMENT.

Per Annum, 8s. 6d., post free.

Monthly Parts, 6d.; post free, 8d.

Ditto per Annum, 7s. 6d., postfree.

### THE HOSPITAL

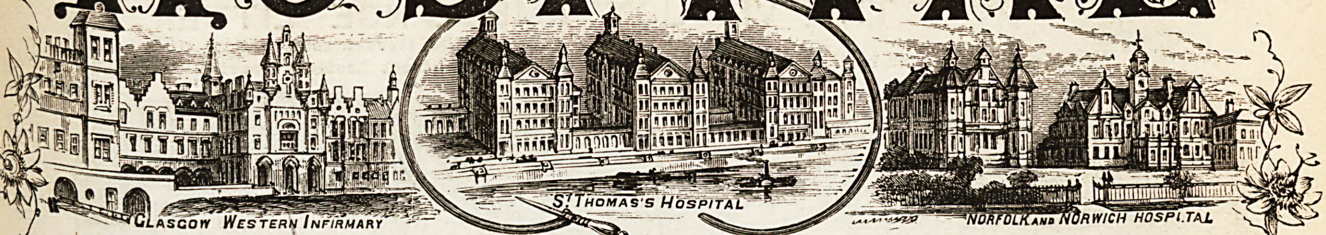

WITH EXTRA NURSING SUPPLEMENT.
No. 288. Vol. XII.] SATURDAY, APRIL 2nd, 1892. [TWOPENCE.

